# Feeding Children and Maintaining Food Service Operations during COVID-19: A Mixed Methods Investigation of Implementation and Financial Challenges

**DOI:** 10.3390/nu13082691

**Published:** 2021-08-03

**Authors:** Erica L. Kenney, Caroline G. Dunn, Rebecca S. Mozaffarian, Jane Dai, Katie Wilson, Jeremy West, Ye Shen, Sheila Fleischhacker, Sara N. Bleich

**Affiliations:** 1 Department of Nutrition, Harvard TH Chan School of Public Health, 665 Huntington Ave, Boston, MA 02115, USA; rmozaffa@hsph.harvard.edu; 2 Department of Social and Behavioral Sciences, Harvard TH Chan School of Public Health, 677 Huntington Ave, Boston, MA 02115, USA; 3 Department of Health Policy and Management, Harvard TH Chan School of Public Health, 677 Huntington Ave, Boston, MA 02115, USA; cdunn@hsph.harvard.edu (C.G.D.); jdai@hsph.harvard.edu (J.D.); ye_shen@fas.harvard.edu (Y.S.); sbleich@hsph.harvard.edu (S.N.B.); 4 Health Resources and Services Administration, U.S. Department of Health and Human Services, Washington, DC 20201, USA; 5 Urban School Food Alliance, 1612 K Street NW, Suite 200, Washington, DC 20006, USA; kwilson@urbanschoolfoodalliance.org (K.W.); jwest@urbanschoolfoodalliance.org (J.W.); 6 Georgetown University Law Center, 600 New Jersey Ave, NW, Washington, DC 20001, USA; sheilafly9@gmail.com

**Keywords:** school meals, food insecurity, school food service authority, costs, COVID-19, implementation strategies

## Abstract

The United States Department of Agriculture (USDA) National School Lunch and Breakfast Programs are critical for the health and food security of U.S. schoolchildren, but access to these programs was disrupted by COVID-19 pandemic-related school closures in spring 2020. While temporary policy changes to the programs enabled school food authorities (SFAs) to pivot towards distributing meals throughout their communities instead of within school buildings, SFAs faced complex challenges during COVID-19 with minimal external support. This mixed methods study investigates the implementation and financial challenges experienced by twelve of the largest urban SFAs in the U.S. during COVID-19. We conducted semi-structured interviews with SFA leaders and analyzed alongside quantitative financial data. We found that SFAs reconfigured their usual operations with nearly no preparation time while simultaneously trying to keep staff from contracting COVID-19, accommodate stakeholders with sometimes competing priorities, and remain financially solvent. Because student participation was much lower than during regular times, and revenue is tied to the number of meals served, SFAs saw drastic decreases in revenue even as they carried regular operating costs. For future crises, disaster preparedness plans that help SFAs better navigate the switch to financially viable community distribution methods are needed.

## 1. Introduction

Ensuring that children in the United States (U.S.) have continuous access to nutritious foods that support proper development is a critical public health goal. Yet, the nutritional quality of U.S. children’s diets tends to be poor overall, and it is a particular concern for children in lower income households, who are at highest risk for poor nutrition and food insecurity [1,2,3,4,5].

Two key programs that protect school-aged children’s nutritional health and food security are the U.S. Department of Agriculture National School Lunch (NSLP) and School Breakfast Programs (SBP), which can provide over half of a school-aged child’s daily nutritional intake [6]. Administered at the federal level, NSLP and SBP facilitate schools’ provision of healthy lunches and breakfasts by providing reimbursements when USDA nutritional requirements are met [7]. School food authorities (SFAs), which are typically financially and operationally separate entities from the school districts themselves, hold the responsibility for sourcing, purchasing, planning, and providing meals, and rely almost entirely on these USDA reimbursements for their operating budgets. The reimbursements are designed to cover the full or partial cost of a meal for children in households at or below 185% of the federal poverty level, meaning they qualify for free or reduced-price meals (FRPM), and slightly offset the costs of meals for children who are above this income threshold and are expected to pay full price [7]. Nearly 30 million U.S. youth participate in NSLP and over 17 million participate in SBP; about 75% of these youth qualify for FRPM [8,9]. Particularly for children who qualify for FRPM, these programs reduce food insecurity, improve diet quality, and promote healthy weight [10,11,12,13,14,15,16,17].

However, starting in the spring of 2020, coronavirus disease 2019 (COVID-19) related school closures severely disrupted these essential nutrition programs [18], and food insecurity among children increased dramatically, particularly for Black and Hispanic/Latinx families [19]. To facilitate continued access to school meals during school closures, individual school districts tried to continue serving meals, and to facilitate this, Congress authorized the USDA to: (a) establish the Pandemic-Electronic Benefit Transfer (P-EBT) program, which aimed to distribute EBT cards to FRPM-eligible students loaded with the cash value of the missed school breakfasts and lunches to enable purchasing food in stores (although this was not fully implemented in many states for several months after authorization); and (b) waive certain NSLP and SBP requirements to allow districts more flexibility in distributing meals while mitigating disease risk. These waivers permitted SFAs to serve: multiple meals at once, meals outside of a “congregate” or group setting (e.g., a cafeteria), and, eventually, meals free of charge to all youth under 18 regardless of their FRPM status [20]. This was a critical development, as prior to the COVID-19 pandemic, only SFAs that participated in the Community Eligibility Provision (CEP) were able to serve meals free of charge to all students. CEP allows for the serving of free meals, via a special financial arrangement between USDA and participating school districts, to all students in a school and/or school district (regardless of individual students’ own family income status) based on area-level income eligibility [21].

Despite the critical role SFAs played in distributing school meals in the community during COVID-19 school closures, little is known about the implementation process the SFAs used and how it impacted their finances and operating procedures, though initial reports suggest substantially fewer meals were served throughout the pandemic, translating to an estimated 30% reduction in meals served nationwide [22]. Understanding how SFAs approached implementing school meal programs during COVID-19 school closures, and identifying critical operational and financial support for successful implementation, is essential for informing strategic planning for continuing school meals programs during future crises [23].

Using a mixed methods approach guided by the Consolidated Framework for Implementation Research (CFIR) [24], the purpose of this study was to (1) estimate the impact of COVID-19 school closures on school food service costs, revenue, and ability to serve meals in large urban school districts; and (2) gather SFA administrators’ perspectives on implementing school meal distribution programs during school closures and reopenings. Academic researchers partnered with the Urban School Food Alliance (USFA), which is an organization of 12 of the largest SFAs in the country, whose members collaborate to share best practices, develop procurement strategies, and advocate for the health and wellness of the 3.2 million students they serve.

## 2. Materials and Methods

### 2.1. Study Design

This mixed methods, concurrent triangulation study [25] was conducted between October and November 2020 and consisted of (1) in-depth interviews with food service directors from the 12 districts participating in USFA; (2) a review of detailed meeting notes from monthly USFA meetings from March 2020 to January 2021; and (3) the collection of quantitative financial and meal count information from those districts. The Harvard T.H. Chan School of Public Health Institutional Review Board approved all study procedures and materials.

### 2.2. Recruitment and Study Population

The 12 districts participating in USFA at the time of the study, and thus participating in this study, were large urban districts from across regions of the U.S.; seven districts were located in the U.S. South, three were located in the U.S. Northeast, and one each were located in the U.S. West and U.S. Midwest. The districts were recruited because of their membership in USFA as well as the potential for FSA leadership in each district to serve as key informants in an investigation into school food service in urban districts. With the assistance of USFA leaders, we invited SFA administrators from the 12 districts to participate in a one-hour virtual interview with the research team. SFA administrators were also invited to include additional district food service staff to provide relevant insights. Twenty participants, representing all twelve school USFA member districts, participated in the interviews between October and November 2020. For each district, the SFA director participated; in six districts, additional food service administration leaders participated. Of these 12 districts, ten provided detailed information on costs for the quantitative study component.

### 2.3. Qualitative Measure Development, Data Collection, and Analysis

#### 2.3.1. Interview Guide Development

The interview guide questions focused on a broad range of implementation activities within five key CFIR domains: (1) intervention characteristics; (2) outer setting; (3) inner setting; (4) characteristics of individuals; and (5) process [24]. Questions were designed to facilitate discussion of implementation changes to NSLP and SBP and perceived needs during the COVID-19 pandemic by describing approaches in a typical school year (i.e., 2018–2019) and drawing comparisons to approaches during spring-fall 2020. Questions were also informed by an existing bank of qualitative questions for school food operations during COVID-19 [26]. Expert stakeholders reviewed and suggested revisions to the guide, and interviewers (CD, RM, JD) debriefed following initial interviews and discussed areas of confusion or duplication with the larger research team. Minor changes to the interview guide were implemented after three (25%) interviews. See Appendix A for a copy of the final interview guide.

#### 2.3.2. Data Collection

A primary interviewer (CGD) conducted semi-structured interviews using a secure video-conferencing platform, lasting on average 64 min (range 58–74 min). A second member of the research team (JD or RM) attended all interviews to assist with notes. All interviews were audio-recorded, transcribed verbatim using a professional transcription service, and reviewed for accuracy (JD).

#### 2.3.3. Qualitative Data Analysis

We used a framework analysis approach [27] grounded in CFIR [24] to understand and synthesize SFA administrators’ perceptions of the implementation process of new strategies for food service during COVID-19 school closures and their impact on financial health. To develop a codebook for analyzing the interviews, CGD and JD reviewed interview transcripts and USFA meeting notes to develop a broad set of deductive codes aligned with CFIR for categorizing content. The coders also used inductive coding approaches to identify additional constructs that emerged from the interviews to tailor CFIR constructs to our unique landscape of COVID-related implementation experiences. To evaluate the clarity and appropriateness of the codebook, CGD and JD double-coded two of the twelve interviews with the initial codebook. Any coding discrepancies were reviewed and reconciled through discussion; all final decisions were approved by the research team. Using the final codebook, JD coded the remaining eight interviews. NVivo qualitative data analysis software (NVivo version 12, QSR International Pty Ltd., Melbourne, Australia) was used to organize coded data. With all the textual data organized, the investigators (CGD, JD, and RSM) then reviewed the text, sorted by code, using an inductive approach to identify important themes that had emerged both within and across codes.

### 2.4. Quantitative Component: Measures, Data Collection, and Analysis

We obtained SFAs’ revenue and cost data for three different time points: the 2018–19 school year (pre-COVID); spring 2020 (March–June 2020, during COVID closures); and fall 2020, when some districts re-opened for in-person or hybrid learning. For each time point, revenue data captured estimated meal reimbursements, vending/a la carte sales, and grant funding and other donations. We collected cost data using a framework from the Childhood Obesity Cost Effectiveness Study, as well as standard costing protocols [28,29] for labor, meals, equipment/materials, food losses (due to spoilage or waste), and donations of food from SFAs to other organizations in the charitable food system.

#### 2.4.1. Cost and Revenue Data Sources

Data on revenue, cost, and program reach came from several different sources. First, USFA had previously collected revenue, cost, and reach (i.e., meals served) data for the 2018–19 school year from 10 of the 12 districts through their technical assistance work, and provided this information to the Harvard research team. Next, USFA provided the research team with detailed notes from weekly meetings with member districts from March 2020 to January 2021 that covered information about revenues, costs, and meals served during school closures in spring 2020 and fall 2020. Additionally, the research team reached out to each of the individual districts to request budgetary and administrative data. The semi-structured qualitative interviews and administrative data shared by districts provided additional revenue and cost data. Across these efforts to obtain financial data on expenses and revenues for each time point, we obtained financial data on the 2018–19 school year and the spring 2020 period from 10 of the 12 districts, and further obtained fall 2020 data from 7 of the 12 districts. For the 10 districts with any financial data from spring or fall of 2020, each district’s food service director(s) reviewed our summary of financial data for accuracy and completion.

#### 2.4.2. Quantitative Data Analysis

We obtained sociodemographic information for each district for the 2018–2019 academic year (and the 2019–2020 year when available) from the National Center for Education Statistics [30] and from districts’ websites. To assess the differences between revenues and costs experienced by SFAs at each time point, we calculated the total revenue and expense per district and per meal. For each time point, we also calculated the percent contribution of each cost category (food, labor, equipment, and food losses) to the total cost per meal. The average number of meals served per student (all students, not only FRPM-eligible) at each time point was calculated by dividing the districts’ reported average weekly meal counts for each time period by the total number of students enrolled in each district. For each indicator across all districts, we calculated the median and range to account for skewed distributions.

#### 2.4.3. Data Triangulation

To integrate the qualitative and quantitative findings, we mapped key themes from each participating district’s interviews regarding the district’s processes for implementing meal service (e.g., delivery versus onsite, fresh versus shelf-stable foods), pre-existing resources, receipt of implementation supports, and relationships with other community entities providing meals and other services during the COVID-19 pandemic, sorted by district. We then combined these key theme summaries with the quantitative estimates derived for each district of labor cost per meal, food cost per meal, equipment cost per meal, difference between revenue and expense per meal, and difference between pre-COVID and during-COVID numbers of meals served per week per student. With these data aligned by district in a tabular form, all team members reviewed the data to explore whether linkages or patterns between implementation factors and financial health were apparent.

## 3. Results

### 3.1. Sample Description

The 12 districts in our sample served racially and ethnically diverse student bodies (Table 1). Each district served a median enrollment in 2018–2019 of over 200,000 students across a median of 244 schools. Many schools in these districts participated in CEP and had very high levels of eligibility for FRPM.

**Figure 1:**
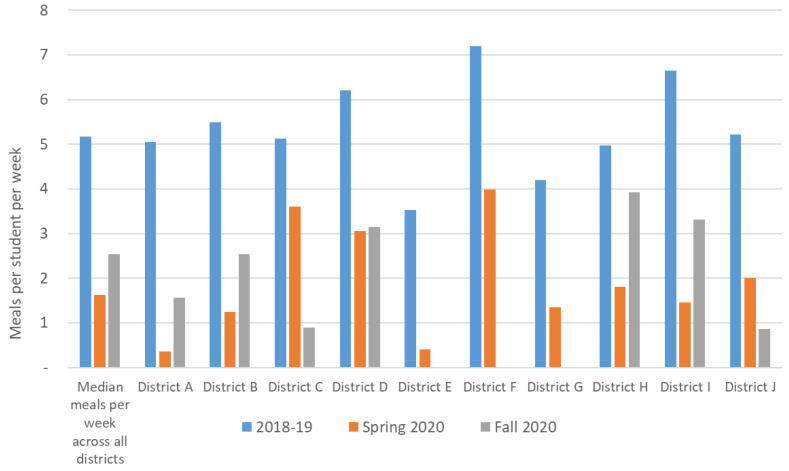
Average and district-level meals per week per student across 10 urban school districts during traditional in-person; Spring 2020 during school closures due to COVID-19; Fall 2020 during remote or hybrid learning. *N =* 10 districts 2018-19 and Spring 2020 during school closures due COVID; *N =* 7 districts Fall 2020.

Consistent with district size, SFAs served a large population of students during regular operations in SY 2018–2019 (Table 2, Figure 1). On average (median), districts served 1,005,888 meals per week (range 302,857–4,750,000]; this translated to about 5.2 (range 3.5–7.2] meals served per week per student. In Spring 2020, with widespread school closures due to the COVID-19 pandemic, the number of meals served per week dropped precipitously, to a median of 318,190 (range 28,419–1,975,886], or 1.6 (range 0.4–4.0] meals per week per student. In Fall 2020, numbers of meals served increased again for some districts, particularly districts where there was a return to in-person school at this time, but remained low for others that continued some sort of remote school.

### 3.2. Qualitative Findings

Findings from the qualitative analysis of school meal implementation during the COVID-19 pandemic are presented by theme below and in Table 3.

#### 3.2.1. Theme 1: Serving Meals during COVID-19 Was a Highly Complicated Process

School meal service during school closures was a complicated system that called for adaptive responses. SFA leaders described rapidly making an enormous number of decisions, based on little concrete guidance, to prevent disruption to meal service for students in need. Several had to figure out within the span of one weekend how to completely shift their standard operations of preparing fresh food to serving shelf-stable food that could be transported without refrigeration. They also had to develop strategies for distribution, trying to determine optimal sites for meal pick-up or identifying routes to deliver meals throughout neighborhoods. Across the 12 districts, all 12 reported setting up sites for “grab and go” curbside meal pickups during school closures; five of these districts also tried to offer meal delivery services in their communities. Six of the districts reported sticking with the same curbside procedures throughout school closures, while six reported troubleshooting and trying different options for meal distribution (e.g., adding or consolidating meal sites, trying out delivery services). Several participants also noted that parents/caregivers’ concerns about neighborhood safety were a significant challenge for determining meal distribution options. Additionally, in some districts where schools reopened intermittently during the pandemic, SFAs had to develop up to three different, but concurrent, modes of delivering meal to students attending school on-site and remotely. This was a significant departure from typical school meal service, where operations were determined months in advance and students primarily received food in cafeteria-style, in-person settings.

Fear of COVID-19 transmission. These restructuring strategies occurred in the context of the fear of contracting COVID-19, and, initially, a lack of information about how it was transmitted. In spring 2020 when SFAs were first undergoing the process of restructuring their operations, there was no precedent in developing meal service models that kept staff safe. Several participants reported their kitchens were too small to have enough staff preparing food at the same time while adequately distanced, and several also described the challenges of setting up safety protocols for meal pick-up and delivery at distribution sites. These challenges regarding exposure to COVID-19 were of particular concern for older staff and staff of color.

#### 3.2.2. Theme 2: The Usual Financial Model of School Food Service Is Untenable during School Closures

All participants described how the typical financial model for school food service, where SFAs depend almost entirely upon reimbursements for each meal served for their operating budget, was inadequate for keeping food service financially solvent and effective during school closures. Given that SFAs continued operating during the pandemic and, in most cases, were prohibited by city leadership from reducing their staffing levels, this meant that SFAs’ labor expenses were the same as pre-pandemic expenses. SFAs also took on extra food costs in many cases, such as more expensive shelf-stable items (e.g., ultra-pasteurized milk) or pre-prepared boxed lunches in order to meet community needs. However, while labor costs remained the same and food costs increased, revenue was much lower due to sharply decreased meal service—and thus fewer reimbursements—from the school closures. SFA leaders reported this as substantially affecting their operations, as explained by one study participant:

*“I’ve been in the hospitality business now 41 years. And anybody who’s been in that business, you understand that [if] you control your food and your labor, you’re good. If you don’t have a control on them, you’re toast….our labor cost is extremely high and our reimbursement is still the same.”*

Before the congressionally authorized USDA waivers to serve all children regardless of income eligibility went into effect, SFAs in districts without CEP described the logistical (e.g., maintaining social distance) and financial burden (e.g., license-plate checking apps) of developing protocols to check eligibility. After the waiver, these non-CEP districts were able to provide free meals to all students without going through a tedious, and potentially risky for COVID-19 transmission, process of identifying the FRPM eligibility of each student coming for a meal. In contrast, SFAs that participated in CEP reported that they had a much smoother experience, as noted by one participant:

*“This is one of the instances in which the benefits of CEP… showed itself because we did not have to engage in the theatrics of accountability and just ridiculous compliance exercises that other districts had to do at a time when you’re trying to minimize interaction between people during a crisis.”*

#### 3.2.3. Theme 3: The Existing Culture of School Food Authorities Sustained Morale throughout the Pandemic

Commitment to fighting hunger. A key aspect to the successful implementation of school meal distribution during COVID-19 was the SFA leaders’ and staff members’ commitment to protecting food security. Study participants referred to their own and their colleagues’ commitment to and passion for providing food for everyone in need. This community- and service-oriented mentality helped motivate SFA leaders to persist in trying new ways to feed families during the pandemic:

*“We’re here for kids and we’re going to take care of our kids, whether they’re in school or not in school, whether school was open or it’s closed… We have a responsibility here and an obligation.”*

Demonstrated grit by underappreciated food service staff. This commitment to addressing food insecurity was highlighted also in the context of SFA directors’ concerns about the wellbeing and low pay of their staff. Several study participants noted that their team members’ commitment to coming into work was inspirational, given widespread fear of COVID-19 transmission. In addition, participants described how their staff were not being recognized as “essential workers” or heroes in the same way that other essential workers at the time (e.g., medical professionals) were valorized.

#### 3.2.4. Theme 4: External Policies and Factors That Influenced Implementation

Poor coordination and communication at multiple levels of government. Across districts, participants consistently described navigating a complicated, and often changing, set of parameters for implementing school meals during COVID-19. SFA administrators were faced with initially little guidance from USDA, public health agencies, and state and local governance regarding reopening schedules and best practices for socially distance meal service. If and when provided, this guidance was often released rapidly, with little time for SFAs to plan how to comply. There were also conflicting opinions and preferences from district superintendents, USDA, and local community members regarding meal composition. For example, one participant reported that their city’s mayor announced at a press conference that the schools would provide meals to anyone in the community, free of charge, without first speaking with SFA leadership, requiring the participant to rapidly try to figure out how to estimate demand and how to cover the costs of meals for non-students, which are not reimbursable. These uncertainties made it difficult to control and predict costs, especially for districts who faced penalties for violating contracts with the vendors that supplied foods and ingredients and were thus locked into paying for their pre-COVID food supply. Participants also reported that while the USDA waivers allowed for flexibility in how meals were served, they were not necessarily effective in helping SFAs reach more families. As one example, there was limited to no guidance in the initial stages of the COVID-19 pandemic on where to set up distribution sites, how many to set up, or whether to use mobile delivery systems, among other key areas of technical assistance needs. Additionally, while some districts reported prior experience with disruptions to school meal service due to weather-related disasters, they also noted that their pre-existing guidance for disasters was inadequate for addressing the longer-term disruptions due to COVID-19.

School meals are part of a safety set that includes other programs. As other community organizations, such as food pantries or churches, worked to quickly distribute food, there was little coordination with SFAs. While increased access to free food was important for alleviating food insecurity, these alternate sources of meals became something of a financial challenge for SFAs given their dependence on keeping meal uptake high for USDA reimbursements. One district noted that they had difficulty distributing meals because so many other local organizations were doing the same thing but with fewer restrictions on what and whom they could serve (i.e., they could buy new food and pivot to serving the types of foods community members liked; there were fewer restrictions on who could obtain the meals). Study participants reported that this, in addition to the rollout of the (P-EBT) program as a separate mechanism for distributing emergency relief to families with FRPM-eligible students, was helpful and necessary for preventing hunger in the community, but further depressed their revenue and ability to maintain financial solvency.

Learning Communities. A supportive resource from the outer setting that participants referenced was the collaborative, Learning Community-style structure of coalitions where SFA leaders could share resources and best practices with their peers:

*“One of the most important elements was the Urban School Food Alliance and our peer school districts. We did a lot of sharing of best ideas, best approaches, what works, what doesn’t work. And so we were really able to learn from each other’s experience and really kind of kind of, you know, raise the boat for children across the entire country in our respective cities and school districts.”*

### 3.3. Quantitative Findings on Financial Health

In school year 2018–2019, prior to the COVID-19 pandemic, districts’ expenses for operating school meals programs were roughly equivalent to the amount of reimbursement they received, allowing most districts to operate without debt, although some districts did have slightly higher expenditures compared to revenue (Table 4). The median expense per meal (including labor, materials/equipment, and food costs) across the districts was USD 3.21 (range 2.38–4.32), and the median revenue per meal, coming entirely from NSLP and SBP reimbursements, was USD 3.11 (range 2.75–3.21).

**Figure 2:**
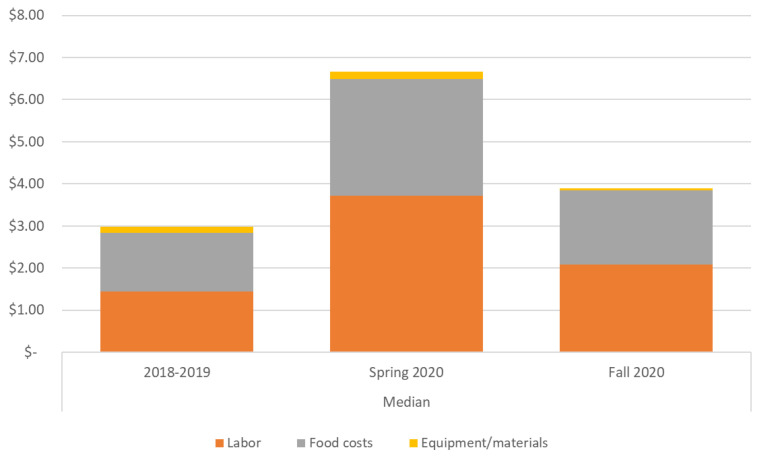
Median per meal expense contributions by expense category across 10 urban school districts during traditional in-person; Spring 2020 during school closures due to COVID-19; Fall 2020 during remote or hybrid learning. *N =* 10 districts 2018-19 and Spring 2020 during school closures due COVID; *N =* 6 districts Fall 2020.

However, in spring 2020 as schools closed, this balance between expenses and revenues was lost. While revenues per meal increased slightly, partly due to districts receiving some philanthropic donations to offset costs and partly due to slightly higher reimbursement levels for districts that opted to offer meals through the USDA Summer Food Service Program (SFSP), expenses per meal increased dramatically, to a median cost of USD 6.61 per meal (range 2.92–58.76). This increase in expense per meal was driven by multiple factors. First, producing school meals for delivery and maintaining equipment and protocols to keep food service workers and students safe from COVID-19 cost more than usual times. Districts saw small equipment expense increases due to the need to purchase personal protective equipment (PPE) and some increases in food costs as districts moved towards purchasing different types of foods more suited to distribution (e.g., shelf-stable milk) (Figure 2). Second, some districts took on the additional expense of providing meals to community members regardless of age–so they served more meals, without reimbursement through the NSLP or SBP (as these programs will only reimburse for meals to students in that district). A third, and possibly most salient, factor was the fact that most districts maintained pre-COVID staffing levels; although some districts saw workers quit or retire during the pandemic, districts could not lay workers off and instead kept them on payroll. This resulted in a situation where districts had labor costs that were not proportional to the number of meals being served.

Thus, although there were some increases in food and equipment costs, it appeared that the mismatch between per meal expenses and revenue was largely driven by drastic decreases in uptake of the meals even while districts had to maintain the same operating costs as pre-COVID.

**Figure A1:**
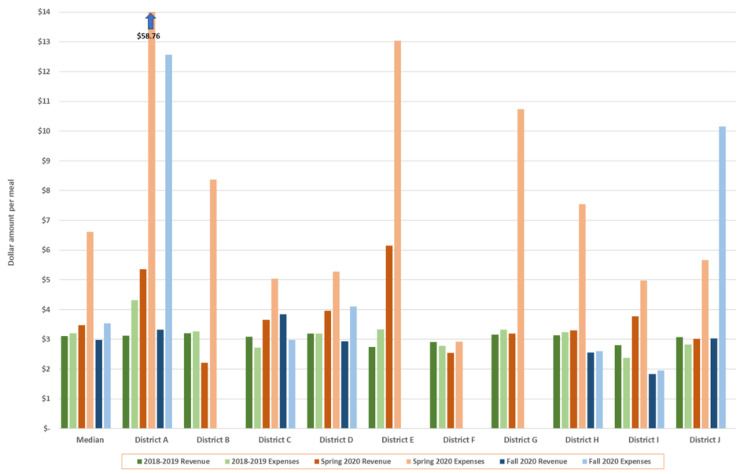
Median and district-level per meal revenue and expense across 10 urban school districts during traditional in-person; Spring 2020 during school closures due to COVID-19; Fall 2020 during remote or hybrid learning. *N =* 10 districts 2018-19 and Spring 2020 during school closures due COVID; *N =* 6 districts Fall 2020.

Although several districts began hybrid (i.e., partially in-person) learning in Fall 2020, meal uptake remained low, especially for districts that maintained remote learning in Fall 2020. For these districts in particular, the gap between expenses per meal and revenue widened, worsening the school food service programs’ financial situation. District-level per meal revenue and expense at all three time points are presented in Appendix B (Figure A1).

### 3.4. Concurrent Triangulation of Qualitative and Quantitative Findings

**Figure 3:**
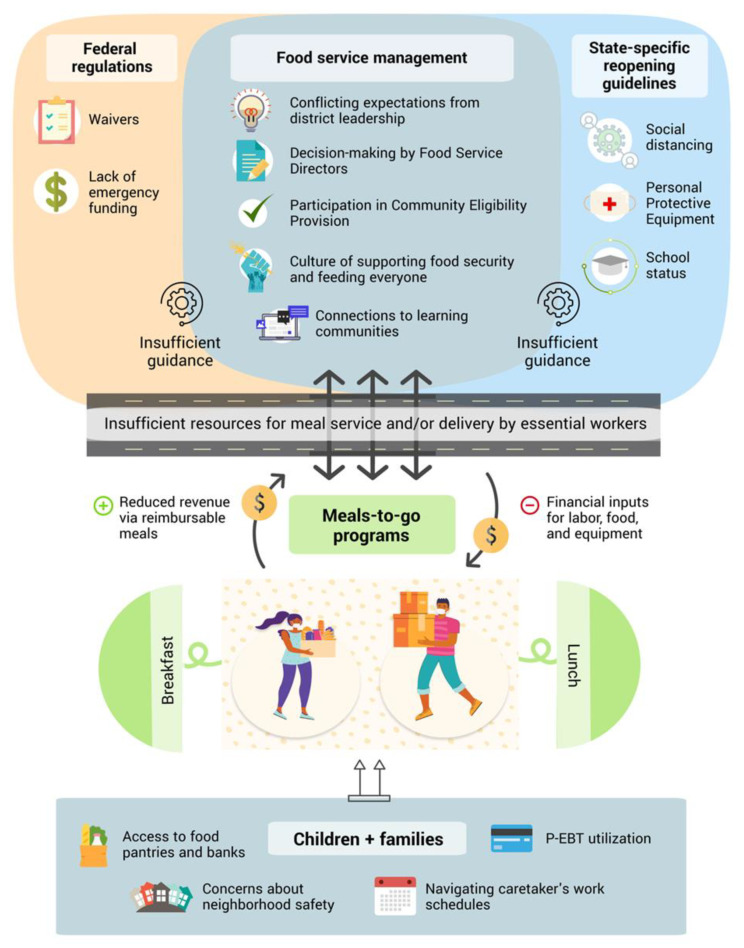
Visual map of program implementation and financial challenges faced by school food authorities (SFAs) during COVID-19 using qualitative and quantitative data.

Several themes raised in the qualitative interviews aligned with the quantitative cost data. Maintaining pre-COVID operating expenses in the face of post-COVID declines in meal uptake put districts at a financial disadvantage; this was reflected in participants’ accounts of the challenges in maintaining salary and benefits for food service workers while working with reduced revenue. Another theme that resonated across both sets of data was the impact of concurrent community initiatives to distribute meals. Districts that reported substantial meal distribution from community relief organizations had much lower meal uptake during COVID-19 compared to districts that did not report this as a strong issue, and that these districts subsequently had substantially larger costs per meal given the low number of meals. Finally, districts that reported other significant challenges with monitoring and distributing meals during school closures, such as community concerns of crime, also showed lower meal counts and subsequently higher costs. Altogether, using both qualitative and quantitative data, Figure 3 illustrates the complexity and financial challenges that SFAs faced during COVID-19 school closures.

## 4. Discussion

In this mixed methods study, we found that, during COVID-19 school closures, SFA leaders and their staff were faced with an incredibly complex challenge while operating under a financial model that did not match the moment. SFAs quickly shifted operations to ensure students were food secure, with little context-specific guidance and no precedent to follow. At the same time, they were required, due to local level policies, to maintain their pre-pandemic expenses, but were receiving drastically fewer reimbursements and minimal other forms of supplemental financial support. As a result, districts now face enormous financial challenges. Our findings highlight the need for a reconsideration of the funding structure for school meals. Ensuring that these meal programs are given the implementation supports they need to succeed is crucial.

SFAs were tasked with rapidly innovating and shifting their implementation model from regular school-based meal service to essentially adopting a community-based charitable food distribution model (i.e., food pantries). [18] However, unlike other charitable food organizations, which tend to operate based on grant funding and philanthropic donations, and also may use more volunteer labor as opposed to paid labor, SFAs operate more like restaurants, where their financial viability depends on high uptake of meals so that they can get paid for each meal served. Since meal uptake plummeted during school closures–despite SFAs’ best efforts, it was impossible to keep their reach at pre-pandemic levels, particularly in areas where families were not able to easily access the meals due to parental work schedules or neighborhood safety concerns—this left SFAs in a financially untenable position, and unable to gather enough revenue to offset the costs they were locked into bearing. Particularly during emergencies such as these, but also potentially during regular operations, the financial model for school meal programs should be reconsidered. Given school meal programs’ critical role in the federal nutrition safety net, these programs should be reconceptualized. One approach might be to move from a restaurant-style model to one more similar to other federal nutrition safety net programs such as the USDA Special Supplemental Program for the Nutrition of Women, Infants, and Children (WIC), which does not require individual WIC agencies to operate their budgets based on how many families fully redeem their WIC benefits.

Our findings, especially those related to the complexity of switching to school MTG and the need for strong communication between different actors within the emergency food system, are consistent with other recent investigations [31,32,33,34]. These studies also suggest a need for a comprehensive disaster management plan for nutrition and charitable food programs. Our findings suggest that such a plan would need to identify how to coordinate responses across all stakeholders involved in emergency feeding, so that it is clear who will do what in terms of distributing meals and cash benefits for food during future crises. During COVID-19, the fact that multiple organizations were trying to distribute food and cash benefits to the same target populations resulted in an unfortunate situation of having SFAs “compete” against charitable food organizations with more flexibility, as well as against P-EBT. Our findings, as well as Patten et al.’s [31], also suggest the importance of including SFA leaders in the development of such plans as key stakeholders, so that those making the decisions about distributing meals are speaking with stakeholders with on-the-ground experience. A cohesive, coordinated approach across multiple modes of food and benefit delivery is needed both to ensure families are supported and to ensure organizations can effect change in an efficient and financial solvent manner.

Immediately adopting a universal school meals (USM) approach may be a critical first step for school meal programs during an emergency, given that districts with CEP reported much more seamless transitions to school MTG and safer working conditions for food service employees with this approach. USM could also have additional economic benefits to programs [35] and substantial health and academic benefits to students [36,37].

Strengths of this study include the partnership between USFA and an academic research institution to obtain rich, first-hand accounts from SFA leaders—those involved most closely with operating school meal programs during the COVID-19 pandemic—as well as obtaining detailed information on SFA finances. An additional strength was the information shared and advice given through an ad hoc COVID-19 working group supported jointly by Healthy Eating Research (HER), a national program of the Robert Wood Johnson Foundation, and the Centers for Disease Control and Prevention supported Nutrition and Obesity Policy Research and Evaluation Network (NOPREN) who were simultaneously conducting research on schools. Limitations include our small and unique sample of districts, which, while representing 12 of the largest school SFAs in the country, may not be generalizable to other urban districts, as well as districts in suburban or rural settings. However, as noted above, our findings were consistent with and complementary to findings from two other recent studies involving interviews and surveys with school food service staff in different samples [31,32]. Another limitation was that we were unable to collect some financial data from all 12 of the recruited districts. It is possible that the districts with missing financial data had substantially different experiences related to the discrepancies between costs and revenues; notwithstanding, the included districts still showed a relatively wide range of experiences, and our results were consistent with a recent survey of school food finances conducted in a larger and more diverse sample of U.S. schools (Kenney et al. 2021, unpublished).

## 5. Conclusions

School food service programs are a linchpin of the federal nutrition safety net, providing crucial nutritional support to tens of millions of US youth. However, the current operations structure is not responsive to disaster. To build in flexibility and ensure children’s access to meals is not disrupted both during future emergencies and regular operations, Congress should consider funding these programs more like other agencies that carry out other nutrition safety net programs like WIC, rather than requiring school food service programs to rely solely on per-meal reimbursements for revenue, which forces them into a situation where they cannot recoup their costs during emergencies. Additionally, emergency preparedness plans that incorporate the voices and perspectives of SFA leaders and involve coordination between SFAs and other nutrition safety net organizations need to be developed [34]. More work remains to learn what worked and what did not during COVID-19 child nutrition feeding program adaptations so that our country, as well as others, can be better prepared for future disruptions to these programs that may arise from future pandemics or other disasters.

## Figures and Tables

**Table 1:** Demographic characteristics of 12 Urban School Food Service Alliance members, 2018–2019.

	**Median (Range)**
Number of students enrolled, 2018–2019	192,278 (55,214; 1,130,000)
Number of students enrolled, 2019–2020 ^1^	190,014 (51,433; 1,131,868)
Total number of schools in district	242 (118; 1841)
Elementary	143 (73; 800)
Middle	35 (6; 400)
High	35 (20; 330)
	**Mean% (±SD)**
Schools participating in Community Eligibility Provision ^2^	72.3% (40.1)
Students qualifying for free/reduced price meals ^2^	79.8% (15.5)
Race/Ethnicity of students ^3^	
White, non Hispanic	16.5% (8.0)
Black, non-Hispanic	36.0% (19.9)
Hispanic	39.3% (20.1)
American Indian/Alaska Native	0.1% (0.08)
Asian	4.6% (3.4)
Native Hawaiian/Other Pacific Islander	0.0% (0.1)
Other race	0.6% (0.4)
Two or more races	2.8% (1.0)

^1^ *N* = 10 districts. ^2^ *N* = 11 districts. ^3^ National Center for Education Statistics, 2018. https://nces.ed.gov/programs/edge/TableViewer/acsProfile/2018 (accessed on 5 January 2021).

**Table 2:** Median (Range) number of meals served across 10 urban school districts nationwide: 2018–2019 during traditional in-person; Spring 2020 during school closures due to COVID-19; Fall 2020 during remote or hybrid learning.

	School Year 2018–2019		Spring 2020		Fall 2020	
	Median	Range	Median	Range	Median	Range
Total student meals served per week ^1^	1,005,888	(302,857; 4,750,000)	318,190	(28,419; 1,975,886)	318,424	(104,530; 808,210)
Breakfast	313,235	(108,000; 1,657,583)	159,517	(13,803; 844,506)	112,733	(37,697; 248,994)
Lunch	601,065	(159,429; 2,944,444)	158,673	(14,616; 905,814)	147,308	(37,780; 357,425)
Snack	20,429	(0; 277,778)	0.0	(0; 287,773)	22,650	(0.0; 157,323)
Supper	41,317	(0; 375,917)	0.0	(0; 103,458)	25,897	(0.0; 118,459)
Total meals served per week for other community members	0.0	(0; 0)	102	(0; 903,160)	0.0	(0.0; 491,109)

^1^ *N* = 7 districts provided data for total number meals served for Fall 2020; of these, *N* = 6 districts provided data for number of breakfast, lunch, snack, and suppers served for Fall 2020.

**Table 3:** Key themes and illustrative quotations from semi-structured interviews with *N* = 20 food service directors and staff from 12 urban school districts, October 2020.

Theme	Sub-Theme	Illustrative Quotation(s)
Serving meals during COVID-19 was a highly complicated process CFIR Construct: “Intervention Characteristics”	Rapidly setting up school meals-to-go was a complex process	“We were called on a Friday afternoon at about 4:00 and told that the school district was closing and that Monday morning we needed to open up and we needed to be able to feed families. So we had just about 48 h to change our whole method of distribution” “With distribution initially, because it was so sudden we were initially just preparing out of our production site and just curbside. Numbers were really low. We knew that there were people out there, students out there that couldn’t get to the food, so we increasing it. We added bus routes. I think we initially started with one-hundred and twenty-eight, what we called distribution points… trying to find those communities where there was a high density of students that we might be able to reach”
	Complexity of keeping staff COVID-safe	“Like they are low paid, right. Which is not it’s not a job that people like fight to get into, but they have like really showed up and really been scared…. they are really like unsung heroes.” “The first part was really trying to pivot and get prepackaged meals that we could then package in bundles to hand out every day. We couldn’t even pivot to the once a week or two to three day model because the logistics of the volume of it was just too high. We either have to double or triple the amount of sites open in order to get the volume to do more packaged in fewer days. And that we couldn’t do. Because at the time, it was just too risky to have that many staff members working and wanted to limit our exposure”
Theme 2: The usual financial model of school food service is untenable during school closures CFIR construct: “Intervention Processes”	Delivering meals was simpler–and COVID-safe–for CEP schools	“Having the experience feeding CEP and feeding everyone already no charge made it a much easier transition for us than like a typical district where you would have your free, reduced and paid. And for a long time, you know, the policy was no you’re paid, you still have to pay, which was insane. And finally they changed that. So, you know, that was a nice piece of continuity to be able to continue to offer all meals at no cost to all students. Yeah, that that was a positive.”
	Financial model, depending on reimbursements, does not work during emergencies	“Funding needs to be probably looked at a little differently. You kind of got your regular mode of what you going, like if you have a captured audience, but if you don’t have a captive audience when it’s crisis emergency mode, we got to be able to pivot quickly to say we just need to you need to fund us for our expenses. I mean, obviously, we’re going to try with the help of whoever else. Right. But this current per meal model is awful. It’s awful for this crisis. It just doesn’t work.” “You know, I just give a quick reference, I’ve been in the hospitality business now 41 years. And anybody who’s been in that business, you understand that you control your food and your labor, you’re good. If you don’t have a control on them, you’re toast. Having said that, you know, our labor costs in our district are high. You might be aware that all our Part-Time workers are also fully benefited. So our labor cost is extremely high and our reimbursement is still the same.”
Theme 3: The existing culture of School Food Authorities sustained morale throughout the pandemic CFIR Construct: “Inner Setting”	Culture of commitment to fighting hunger	“And so I wouldn’t say that the financial considerations they’re, they’re being considered. But at that moment it was, how do we make our meals accessible to the children and the families of our community?” “You’re just like, we’re going to work through this. I have a positive attitude, right. We going to work through it. I don’t know how yet. But the thing is we’re going to feed kids and we don’t want to turn people away.” “But I’m like we always feed. So it was just I mean, granted, we pivoted quickly, but we always feed and it’s kind of always our mindset… hey, we’re not going to turn families away.”
	Demonstrated grit by underappreciated food service staff	“The one thing I will say, you know, I just need to call out our amazing people both here in [district] and I know in school districts all across the country. I’m just you know, I’ve said this one time on an interview and, you know, when I was young, my heroes were like Superman and stuff like that. And now my heroes are are these amazing men and women that just put themselves on the front line in a time where things were a lot more uncertain about how COVID spreads and and, you know, gearing up to try to take care of kids. And I’m just so proud of the men and women of our department. I just can’t say enough about them. They’re, they’re really my heroes.... And, you know, they, they just hit it. So they hit the nail on the head. You know, we’re here for kids and we’re going to take care of our kids, whether they’re in school or not in school, whether school was open or it’s closed. You know, we have a responsibility here and an obligation. And I’m just so proud of them. And I’ll never be able to say thank you enough for them.”
Theme 4: External policies and factors that influenced implementation CFIR Construct: “Outer Setting”	Poor coordination and communication at multiple levels of government. Some SFAs reported that their city leadership announced that adults could pick up meals at meal distribution sites without telling them first	“[We had] to hold the superintendent back, as they are out there trying to encourage people to come and them to do more, but they have to give him a realistic picture of their capacity” “Even as I, we certainly appreciated USDA making all of these waivers and changes. It was wonderful that we created an ease and a burden for us, but not necessarily reflective of what we, how we could get [to] families.”
	Relationship with other food safety net programs. Pandemic-EBT perceived as beneficial for families, but some districts also perceived that it caused reduced participation in school meals to go, while others perceived no change.	“And then secondly is that families said to me, “We would really love if we could just have more P-EBT funding, which is the pandemic EBT, because we could be able to make the meals that that my kids will recognize and that, that it’s much more convenient and that I can buy what I’m looking for. And then I can also go through like Amazon Fresh or Whole Foods and get it delivered, which is, you guys cannot.” “We actually reduced the amount of distribution sites. We saw towards the end of the spring that the numbers were starting to go down. The P-EBT pandemic card was being, students, families were being informed of that. So that was starting to occur.” “Yeah, I think the P-EBT program, I will also say, has been a good program in my opinion. It seems like families are really benefiting from it and people are trying to take advantage of having it. And I think that’s a great thing for families to have access to. So I hope that that is something that is considered so that if and when this happens in the future, you know, that that’s something that could just be counted on versus like the spotty... It would just be nice to know that that family has had that through the end of the pandemic, at least from that aspect. Fixing our bottom line is a different story.”
	Learning Communities	“We have a coalition, like I said, of about 50 partners, and we meet on a quarterly basis or every, I would say every two months or three months. So it might be four to five meetings a year, so I had those meetings throughout this process and we certainly listened to feedback that we had from our partners. Some of them are parent partners and some of them are educational or organizational partners. We also listened to the Urban School Food Alliance partners very closely. We had an emergency COVID call weekly during the spring and summer. And as a result, we would share best practices and concepts with each other about how we were doing the work.” “One of the most important elements was the Urban School Food Alliance and our peer school districts. We did a lot of sharing of best ideas, best approaches, what works, what doesn’t work. And so we were really able to learn from each other’s experience and really kind of kind of, you know, raise the boat for for children across the entire country in our respective cities and school districts. Just by that that sharing process and benchmarking, we would look at what is somebody’s participation down there in [state] and how are they doing in [district] and [district] and [district] and kind of gave us a sense excuse me, you know, gave us a sense of performance.”

**Table 4:** Median (Range) per meal revenue/expense and percent of total by category across 10 urban school districts during traditional in-person; Spring 2020 during school closures due to COVID-19; Fall 2020 during remote or hybrid learning.

	School Year 2018–2019		Spring 2020		Fall 2020 ^1^	
	Median	Range	Median	Range	Median	Range
Total revenue, weekly	USD 3,137,289	971,429; 15,000,000	USD 1,091,332	141,562; 5,022,718	USD 1,115,780	316,726; 2,068,162
Total expense, weekly	USD 3,119,768	USD 986,221; USD 15,777,778	USD 2,084,734	537,034; 16,450,042	USD 1,314,836	947,713; 2,103,399
Revenue per meal	USD 3.11	2.75; 3.21	USD 3.48	2.21; 6.15	USD 2.98	1.83; 3.85
Reimbursement	USD 3.11	2.75; 3.21	USD 3.20	1.78; 5.43	USD 2.98	1.83; 3.85
Donations received	USD 0.00	0.0; 0.0	USD 0.07	0.00; 1.86	USD 0.00	0.00; 0.00
Expenses per meal	USD 3.21	2.38; 4.32	USD 6.61	2.92; 58.76	USD 3.54	1.95; 12.57
Labor	USD 1.44	1.18; 1.89	USD 3.71	1.71; 42.83	USD 2.08	0.67; 7.39
Food costs	USD 1.40	1.13; 2.32	USD 2.78	0.75; 7.45	USD 1.76	0.62; 4.79
Equipment/materials	USD 0.15	0.02; 0.58	USD 0.18	0.01; 7.45	USD 0.06	0.01; 1.39
Community meal expenses	N/A	N/A	USD 0.00	0.00; 3.70	USD 0.00	0.00; 0.00
Net per meal	−USD 0.02	−1.21; 0.43	−USD 3.45	−53.40; −0.38	−USD 0.65	−9.24; 0.87

^1^ *N* = 10 districts with per meal revenue and expense data for 2018–19 and Spring 2020. *N* = 7 districts with per meal revenue and expense data Fall 2020.

## Data Availability

The data in this study are not publicly available due to the protection of participants’ privacy.

## Appendix A. Qualitative Interview Guide

1. To start, please you tell me a little more about your role in school food service in [DISTRICT]? How many schools do you oversee?
2. Prior to the pandemic, what approach did your district use for preparing and serving meals–do you have a central kitchen from which meals are distributed to schools in the district, or a contract with a larger food service provider (e.g., Aramark, Sodexho), or do schools prepare their own foods in on-site kitchens, or some mixture of these strategies? Something else?
3. Are students in [DISTRICT] currently attending school:
  - Fully in person;
  - Fully virtual/online;
  - In a hybrid model (e.g., some in person, some virtual; half day in person half day virtual); or
  - Something else?
4. Please tell me about your district’s plan to change how students attend school (e.g., switch from fully remote to hybrid once appropriate safety steps are in place).
5. In the USFA meeting minutes, I saw that your district made changes to they way that you were preparing and serving meals in spring, summer, and now [*Research team will populate with examples for each district, taken from meeting notes*]. What implications has this had for your team–have you had to conduct extra trainings, hire or release any staff, pay more overtime, investigate different packaging materials, develop communication materials for families?
  Probe: Are there other major changes I didn’t mention that have had implications for your team?
6. Why did you choose the approaches that you did [*Research team will populate with examples from each district, taken from meeting notes*]? Let’s first start with spring. Now summer. And finally reopening.
  Probe: What went into your decisionmaking process? E.g. financial constraints, community input?
7. How did you and your team assess whether your new approaches were working and what needed to be done differently? Did the transition from summer to fall impact lead to additional changes?
8. Going forward, what new or different approaches to preparing or serving meals will your district keep or start implementing in the future?
  Probe: Why will you continue using these new approaches?
  Probe: What financial impact will these approaches have on your operations?
9. Going forward, what support does your district need in order to continuing feeding children?
  Probe: Can you tell me more about what group or organizations would need to provide this support (USDA, the state, your local community)?
10. Now, let’s talk about key partners. What individuals or groups were consulted when your district made changes to food service operations during spring closures, summer and reopening?
11. Now, let’s turn to challenges of making these changes to school meal service. Please share with us what challenges your district faced in the spring. And any different or unique ones for the summer? And now, this fall, any different or unique challenges?
12. Since the spring closures, please share with any insights related to the needs of students and families in your district to your program during COVID? How has your district tried to address any of these needs? How did student or family input shape your reopening meal service approach?
13. Please discuss further the impacts of the financial or operational challenges you mentioned earlier had on meal service during closures and reopening.
14. Please tell me more about any changes that made it easier or supported your district’s capacity to serve meals during school closures and reopening. Any best practices or lessons learned you would want to carry forward for reopening? Future school closures? Or, in a normal school meal service operation?
15. Please share with us what operational changes could be considered to ensure the financial health of your district’s program, now and in the future.
16. Please share with us how much and what type of financial relief is most needed at this time and in the future?
